# Tribute to Our Reviewers

**DOI:** 10.1002/deo2.111

**Published:** 2022-03-23

The Editorial Board of *DEN Open* takes this opportunity to acknowledge with particular gratitude the following reviewers who have provided their valuable time and expertise in the evaluation of manuscripts submitted to *DEN Open* during the period of November 2020 to December 2021.

Takao Itoi

Editor‐in‐Chief

*DEN Open*

Yasuhiko Abe

Kazuya Akahoshi

Takuji Akamatsu

Ryohei Ando

Akihiro Araki

Kyohei Ariake

Reiko Ashida

Shinya Ashizuka

Shigeki Bamba

Ivo Boskoski

Hideyuki Chiba

Hourin Cho

Akira Dobashi

Osamu Dohi

Shinpei Doi

Shungo Endo

Mitsuru Esaki

Nao Fujimori

Toshio Fujisawa

Mitsuharu Fukasawa

Nobuhiko Fukuba

Shuichi Fukuda

Yoshihiro Furuichi

Osamu Goto

Kenta Hamada

Tsuyoshi Hamada

Noboru Hanaoka

Kazuo Hara

Ryo Harada

Yusuke Hashimoto

Takemasa Hayashi

Mari Hayashida

Susumu Hijioka

Takuto Hikichi

Takashi Hirose

Takahiro Horimatsu

Yusuke Horiuchi

Kinichi Hotta

Takashi Ibuka

Ryoji Ichijima

Katsuro Ichimasa

Toshiro Iizuka

Yuichiro Ikebuchi

Atsushi Imagawa

Hiroo Imazu

Kazuya Inoki

Tadahisa Inoue

Takahiro Inoue

Fumiaki Ishibashi

Yusuke Ishida

Naoki Ishii

Hirotoshi Ishiwatari

Ken Ito

Masahiro Itonaga

Takuji Iwashita

Yugo Iwaya

Shinya Izawa

Tomohiro Kadota

Hideki Kamada

Ken Kamata

Takeshi Kanno

Yoshihide Kanno

Motohiko Kato

Hiroshi Kawachi

Koichiro Kawaguchi

Takaaki Kawaguchi

Noriyuki Kawami

Seiji Kawano

Noboru Kawata

Daisuke Kikuchi

Toshifumi Kin

Satoshi Kinoshita

Shinsuke Kiriyama

Yusuke Kitagawa

Katsuya Kitamura

Hideki Kobara

Hirofumi Kogure

Yoriaki Komeda

Hitoshi Kondo

Yoshiyasu Kono

Yuta Koyama

Shiko Kuribayashi

Yuichiro Kuroki

Takamichi Kuwabara

Toshio Kuwai

Masaki Kuwatani

Kenji Limpias Kamiya

Yasuharu Maeda

Akira Maekawa

Mai Ego Makiguchi

Noriaki Manabe

Tomoki Matsuda

Kazuyuki Matsumoto

Katsuyoshi Matsuoka

Noriko Matsuura

Kosuke Minaga

Hitomi Minami

Yasuhiko Mizuguchi

Hiroya Mizutani

Shuntaro Mukai

Takashi Murakami

Yasuaki Nagami

Mitsuru Nagata

Kazunari Nakahara

Yousuke Nakai

So Nakaji

Jun Nakamura

Masanao Nakamura

Rieko Nakamura

Daiki Nemoto

Manoel Galvao Neto

Ryota Niikura

Keiko Niimi

Tsutomu Nishida

Jun Nishikawa

Toshihiro Nishizawa

Kouichi Nonaka

Satoru Nonaka

Eizaburo Ohno

Shiro Oka

Yutaka Okagawa

Naoki Okano

Mitsuru Okuno

Toru Okuzono

Teppei Omori

Shunsuke Omoto

Manabu Onimaru

Satoshi Ono

Shozo Osera

Yoshihisa Saida

Daisuke Saito

Yoshiki Sakaguchi

Yoji Sanomura

Yu Sasaki

Hiroki Sato

Masau Sekiguchi

Kotaro Shibagaki

Satoki Shichijo

Yusaku Shimamoto

Yuto Shimamura

Masaaki Shimatani

Hideyuki Shiomi

Hironari Shiwaku

Harutoshi Sugiyama

Tetsuya Sumiyoshi

Sho Suzuki

Takuto Suzuki

Kazunori Takada

Kosuke Takahashi

Hiroyuki Takamaru

Yusaku Takatori

Noritaka Takatsu

Yohei Takeda

Mamoru Takenaka

Toshihisa Takeuchi

Naoto Tamai

Yuki Tanisaka

Yutaka Tomizawa

Takeshi Tomoda

Nobuyuki Toshikuni

Yosuke Toya

Takayoshi Tsuchiya

Yosuke Tsuji

Yoshiki Tsujii

Yuki Ushimaru

Yohei Yabuuchi

Atsuo Yamada

Takuya Yamada

Yasushi Yamasaki

Shunich Yanai

Kei Yane

Tomonori Yano

Akira Yoshida

Masao Yoshida

Naohisa Yoshida

Shigetaka Yoshinaga

